# Fall Armyworm Infestation and Development: Screening Tropical Maize Genotypes for Resistance in Zambia

**DOI:** 10.3390/insects13111020

**Published:** 2022-11-04

**Authors:** Chapwa Kasoma, Hussein Shimelis, Mark D. Laing, Bethelihem Mekonnen

**Affiliations:** 1 African Centre for Crop Improvement, University of KwaZulu-Natal, Private Bag X01, Scottsville, Pietermaritzburg 3209, South Africa; 2 Centre for Agriculture and Bioscience International (CAB International) Southern Africa Centre, 5834 Mwange Close, Kalundu, Lusaka P.O. Box 37589, Zambia; 3 Department of Zoology and Entomology, University of Pretoria, Private Bag X20, Hatfield, Pretoria 0028, South Africa

**Keywords:** area under pest progress curve, fall armyworm, host-plant resistance, infestation level, stages of life cycle, FAW rearing, pest reaction type, resistance breeding

## Abstract

**Simple Summary:**

The fall armyworm (FAW) (*Spodoptera frugiperda* J.E. Smith) is now a widespread and resident pest in Africa, where it is causing substantial yield losses to maize. Maize is an important staple food supporting more than 500 million people in sub-Saharan Africa. Since the arrival of FAW to Africa six years ago, multidisciplinary research aimed at managing the FAW and mitigating its impact on food security has been ongoing. Understanding the biology of the FAW under sub-Saharan African conditions and developing simple screening techniques for maize against the FAW will improve research outputs on FAW management. This study aimed to increase the understanding of FAW behaviour under sub-Saharan African conditions and investigated simple methods for the laboratory rearing of FAW and screening of maize. FAW was reared successfully using a natural maize-based diet and an artificial soy- and wheat flour-based diet under specified conditions. The study generated useful information on FAW developmental stages and the reaction types of maize, enabling the identification of promising maize genetics for continued breeding. The baseline information presented in this paper will allow for the controlled rearing, infestation, host screening and integration of candidate FAW-resistant genes into market-preferred maize lines in Zambia and related agroecologies.

**Abstract:**

Knowledge of fall armyworm (FAW) (*Spodoptera frugiperda* J.E. Smith) rearing, infestation and development and precision screening protocols are preconditions for the successful introgression of resistance genes into farmer-preferred varieties. We aimed to determine FAW developmental stages, screen tropical maize and select resistant lines under controlled conditions in Zambia. Field-collected FAW samples constituting 30 egg masses and 60 larvae were reared using maize leaf- and stalk-based and soy- and wheat flour-based diets at 27 ± 1 °C, 60 ± 5% relative humidity and 12 h day length. The resulting neonates were separated into sets A and B. The life cycles of set A and field-collected larvae were monitored to document the FAW developmental features. Set B neonates were used to infest the seedlings of 63 diverse tropical maize genotypes. Egg, larva, pupa and adult stages had mean durations of 2, 24, 20 and 12 days, respectively. Test maize genotypes revealed significant differences (*p* < 0.05) based on FAW reaction types, with lines TL13159, TL02562, TL142151, VL050120 and CML548-B exhibiting resistance reactions, while CML545-B, CZL1310c, CZL16095, EBL169550, ZM4236 and Pool 16 displayed moderate resistance. These genotypes are candidate sources of FAW resistance for further breeding. This study will facilitate controlled FAW rearing for host screening in the integration of FAW resistance into market-preferred maize lines.

## 1. Introduction

The fall armyworm (FAW) (*Spodoptera frugiperda* J.E. Smith) is a highly destructive pest of maize and other major cereal crops [1,2]. FAW is Africa’s newest invasive polyphagous pest, reported in 2016. The presence of FAW in over 44 countries in Africa, combined with existing abiotic and biotic production constraints, is threatening maize production and productivity in the region [3,4]. FAW-inflicted yield losses have risked food security and the livelihoods of over 500 million people who depend on maize production and products [5,6,7]. In sub-Saharan Africa (SSA), FAW causes 21 to 53% yield losses in maize production [8]. Under severe FAW infestations coupled with other abiotic or biotic stresses, yield losses of 80% or complete crop failures have been reported in maize and sweet corn production [9,10].

FAW has severely affected maize and major cereal crop production, food systems and value chains in SSA [11,12]. There is a need for dedicated FAW-resistance breeding programs in Africa to develop and deploy new-generation open-pollinated and hybrid maize varieties. Various FAW management strategies are recommended globally, including the use of biological agents, cultural practices, crop protection chemicals, landscape management practices, transgenic crop varieties, host plant resistance and integrated pest management (IPM). IPM involves a curated combination of more than one of the above methods and is effective, sustainable and environmentally friendly. Breeding for FAW resistance is a core component of IPM for being cost-effective and easy to implement for the farmers who are the end users of the developed technologies [13,14].

FAW-resistance breeding requires the controlled screening of locally adapted, market-preferred and resistant maize genotypes. This will enable gene introgression and the development of high-yielding varieties for an integrated pest control strategy. FAW is a highly gregarious and erratic insect pest, and controlled screening facilities are required for reliably assessing pest development and infestation levels and rating the reaction types of the host to select resistant individuals for breeding. A customised insectarium is required for the mass production of the FAW larvae, while a controlled-environment facility is needed for pest development, infestation, host screening and host selection [14]. Understanding FAW’s growth and developmental stages is crucial to identifying and developing pest management strategies. The life cycle stages of the pest encompass the egg, larva, pupa and moth in that order. The egg stage has a relatively short duration, lasting for a maximum of three days [15]. The larval stage is the most damaging to the host crops owing to FAW’s ferocious feeding activity before the pupation stage, the dormant state of the pest whose duration is dependent on the prevailing temperatures [16].

Screening for FAW resistance can be undertaken in controlled environments or under natural field conditions in hotspot areas. Genotype evaluation in the hotspot areas could lead to the overestimation of resistance due to pest escape rather than host resistance [17]. Escape confounds the selection of desirable resistant genotypes because susceptible individuals can be inadvertently included, rendering a low selection response and increasing breeding cost. Hence, it is vital to undertake complementary controlled screening under greenhouse or screen house conditions with optimal combinations of temperature, relative humidity and day length to enhance the host–pest activity. Both evaluation methods provide complementary data and ensure effective comparisons of the host genotypes under moderate pest pressure. Controlled screening with insect populations from the same larval generation allows for detailed observations of pest progress, host reactions and resistance, ensuring higher selection efficiency. Previous studies documented that pest feeding patterns and the ease of assessing host reactions under controlled screening conditions allow for an improved understanding of the pest–host reaction and pest management conditions [18,19,20].

Temperatures of 24 to 31 °C, relative humidity of 52 to 88% and a day length of 12 to 14 h are reportedly ideal for the controlled rearing of FAW from egg or larval samples collected from maize plants [21,22,23,24,25]. Sampled larvae are grown in petri dishes or glass tubes supplemented with a natural diet composed of fresh maize leaves, stalks or kernels [20,25,26] or an artificial diet composed of vital ingredients including processed flour from soybean, chickpea, wheat germ, maize and castor bean mixed with a sucrose solution [22,24,27,28,29]. Adult moths of FAW are maintained in wire mesh cages supplied with sorghum [24] or maize leaves [22] for oviposition and the perpetuation of the life cycle. Walaa et al. [25] used a FAW feeding solution of 10% sucrose dissolved in water to maintain adult insects.

Due to the relatively recent arrival of FAW in Africa, there is a lack of information on pest initiation and development under local crop production conditions. Additionally, there is no detailed procedure regarding controlled and field-based FAW assessment to guide selection and resistance breeding programs in the region. Gains in FAW resistance breeding programs are dependent on the availability of inexpensive, reproducible and high-throughput methods for pest rearing, infestation and host screening [26,29].

Complementary to the above research efforts, Kasoma et al. [30] screened 253 tropical maize genotypes and developed experimental hybrids that were evaluated under field conditions only. The authors identified promising open-pollinated varieties (OPVs) and single-cross hybrids as foundational maize germplasm for FAW-resistance breeding. The selected lines and hybrids should be rigorously evaluated under controlled environmental conditions and pest pressure for precision phenotyping and recommendation. Knowledge of the rearing, infestation and development of the pest and high-throughput screening protocols are preconditions for successful cultivar recommendation and the introgression of FAW-resistant genes into farmer-preferred and locally adapted maize genotypes. Therefore, the objective of this study was to determine the FAW’s developmental stages and infestation levels, screen tropical maize and select resistant lines under controlled environment and pest pressure conditions for production and breeding in Zambia.

## 2. Materials and Method

### 2.1. Description of the Study Sites

The study was conducted at Mount Makulu Research Station (15°54′83” S, 28°24′81” E with an altitude of 1225 metres above sea level [masl]) in Chilanga district, Lusaka, Zambia. Mount Makulu is the Zambia Agricultural Research Institute (ZARI) headquarters with a national research mandate on cereal, legume, horticultural and oil crops. The site has humid–dry tropical climatic conditions, with sandy loam soils and an average temperature of 22 °C [31]. These agroecological conditions make the site suitable for screening crop germplasm for plant disease and insect pest resistance, including FAW. Since the arrival of FAW in Zambia in 2016, Mount Makulu has had consistent FAW populations and crop damage during the main and off seasons [32].

### 2.2. Mass Production of FAW

#### 2.2.1. Sampling of Eggs and Larvae

Representative samples of FAW constituting 30 egg masses and 60 larvae were collected from Zambia Seed Company Limited research site in Chisamba District (15°22′30.65” S, 28°23′22.23” E, 1251 masl), Central Province, Zambia. Samples were collected using perforated plastic containers from field-grown maize plants of the hybrid ZMS638. Larvae were carefully picked from the leaf whorls of the plants, while fresh eggs were carefully scraped off from the leaf blades and deposited into the plastic containers. FAW eggs were identified following the description of Deole and Paul [33] as small, circular masses of mostly white eggs. Sampled FAW eggs and larvae were grown in petri dishes as outlined below.

#### 2.2.2. Laboratory Procedures

The collected eggs were grown in petri dishes containing a fresh diet of tender maize leaves and left to hatch. After hatching, the neonate larvae were randomly separated into two sets, A and B, and maintained for further use. Using an artist’s paintbrush (synthetic watercolour brush, Jinjiang Jiaxing Groups Co. Ltd., China), some 10–15 neonate larvae were transferred into fresh petri dishes. Neonates constituting Set A were singly transferred into new petri dishes at the third instar to minimise feeding of older larvae on younger and weaker ones.

The larvae were removed from the temporary containers using forceps and transferred into polystyrene petri dishes (100 × 15 mm, Fischer Scientific, United States) supplemented with either a natural or an artificial diet. The natural diet (see Supplementary Figure S1A) consisted of tender maize leaves and young stalks from a local, open-pollinated variety (OPV) ZM 4342. In contrast, the artificial diet (Supplementary Figure S1B) was prepared from processed flour of soybean, wheat germ and other vital ingredients (Supplementary Table S1) and mixed with a 10% sucrose solution prepared from water. The eggs and larvae were grown at temperatures of approximately 27 ± 1 °C, relative humidity of 60 ± 5% and an average day length of 12 h.

The FAW larvae that were collected from the field were grown in petri dishes as described above. Both diets were freshly prepared consecutively to ensure a fresh feed supply for the growing larvae through all the instar stages described below. For instance, the natural diet was replaced every two to three days, while the artificial diet was replaced every four days. Petri dishes were cleaned with a 5% hypochlorite solution to prevent microbial growth between each successive diet change.

The pupae developed from the above larva samples were distinguished as loose, oval cocoons that preceded the mature stage of the FAW [34]. The temperatures and relative humidity during the pupal stage were adjusted to 26 °C and 70 ± 5% using an internal heating system and humidifier, respectively. These conditions were conducive to pupal development [35].

Male and female FAW moths that emerged from the pupa were transferred into cages made of waxy paper. The adult moths in the cage were allowed to mate for subsequent oviposition (Supplementary Figure S2). FAW moths were supplied with a 5% sugar solution by soaking cotton wool balls in a sugar solution and placing these inside the cages on petri dish covers. The fresh eggs of the FAW were carefully scraped off from the surface of the cages using a clean spatula and transferred into new petri dishes possessing tender maize leaves for hatching. New larvae neonates that hatched from the eggs (designated as F1) were separated randomly into sets A and B and maintained as described above for further use outlined below. From the initial 30 egg masses collected at ZAMSEED farm, at least 100 newly hatched larvae per egg mass were generated, providing over 2500 larvae for the study.

#### 2.2.3. Assessing the Survival Rate of FAW Larvae on the Natural and Artificial Diets

Eight sets of FAW larvae (set A), each with 15 neonate larvae, were set aside from a newly hatched egg batch and maintained on a natural diet containing maize leaves and stalks. Another eight sets (set A) from the same egg batch as described above, with 15 neonate larvae in each set, were maintained on the artificial soy- and wheat flour-based diet for screening maize genotypes for FAW resistance. To avoid cannibalism, the 15 larvae making up a single set were placed individually in separate petri dishes, resulting in 15 initial petri dishes for each set. The larvae raised on the two diets were monitored, including the pupal stage. The numbers of surviving larvae for both sets were documented.

#### 2.2.4. Determining the Developmental Stages of FAW

The above larvae samples from set A and the field-collected larvae were monitored to document the metamorphosis and the developmental stages of FAW. Adult insects were allowed to mate and produce a new generation of FAW for further monitoring. The developmental stages of FAW (egg, larva, pupa and adult) and the time required to complete each stage were determined by observing the FAW morphological features and behaviour and counting the number of days taken for each stage.

FAW eggs were identified following the description of [33]. Colour changes of the egg masses from a pale green to grey and finally to black represented the early, mid and late FAW egg stages, in that order.

The earliest larval stage was identified by recording the neonates with shiny blackheads and small bodies. Newly hatched FAW larvae of the earliest stage are known to be positively phototropic and to move by swinging on web-like strings of networks [36]. First instar larvae were identified by their whitish body colour and less shiny heads than the newly hatched larvae. Additionally, the first signs of windowpane feeding damage on a maize leaf indicated the presence of first instar FAW larvae. Second instar larvae were identified by their orange heads, the onset of body colour changes from white to pale green and the observation of big patches of windowpane damage on the maize leaves [34]. Third instar larvae were recorded by their body colour change to green or pale brown and the combined windowpane and pinhole damage. As the third instar progressed, the larvae were hiding between maize leaves and covered in faecal frass [37]. To monitor possible larval–larval transitions, the petri dishes containing fourth to sixth instar larvae were carefully examined for possible signs of moulting, guided by the presence of a FAW exoskeleton [38]. Fourth instar larvae were identified by their dark brown body colour and common leaf-tearing damage. The emergence of prominent body markings, including segmentation, a clear trapezoidal pattern of dots in the eighth abdominal segment and the inverted Y shape on the head served for the identification of fifth instar larvae. Sixth instar larvae were identified by their aggressive feeding that caused leaf tattering and the darkest body colour that made all the body markings observed in the fifth instar most visible.

All morphological characteristics used to identify the different FAW instar stages were confirmed using a compound binocular microscope (VisiScopeSZT360-6, VWR, Italy), following the head capsule width classification described by Montenzano et al. [29] (Table 1).

The prepupal stage was identified by the sudden inactivity of the insect, body compression that defined its segments and the cessation of feeding. The pupal stage was observed as the development of a stiff pupal casing that changed from green to brown as the pupa matured [34]. The moth stage was identified as the emergence from the pupal casing of a winged grey and brown adult insect. Male moths have white triangular patches on the forewings, while female insects are comparatively dull coloured [34].

### 2.3. Screening of Selected Maize Genotypes for FAW Resistance

#### 2.3.1. Genetic Materials

The study used 63 selected tropical maize genotypes comprising 57 elite inbred lines acquired from CIMMYT, four OPVs and two single-cross commercial hybrids as comparative controls (Table 2). The four OPVs are ZM7114, ZM4236, Teost and Pool 16, which are grown mainly by small-scale farmers in Zambia. The two single-cross hybrids are MM501 and MM502, released by ZARI and valued for their resistance to maize streak virus. Out of the 57 CIMMYT inbred lines, 50 were previously selected through rigorous field evaluations in Zambia by Kasoma et al. [32] for their partial resistance to FAW. The 50 lines also have desirable agronomic traits, including grain yield and early maturity.

#### 2.3.2. Experimental Design and Trial Establishment

The 63 genotypes (Table 2) were established in 5 L capacity plastic pots filled with sandy loam soil. The soil was supplemented with 5 g of fertilizer consisting of 8% nitrogen, 18% phosphorous and 15% potassium, and pots were watered to field capacity during planting. The experiment was laid out in a randomized complete block design with three replications. Three seeds, later thinned to two plants per genotype were sown per experimental unit at a depth of 2.5 cm. The pots were watered twice a week to ensure sustained moisture for germination. Emerging seedlings were kept free of weeds.

#### 2.3.3. Seedling Infestation with FAW Larvae

Larvae from Set B were used for the screening of maize genotypes. The first infestation of the maize genotypes with FAW larvae was conducted 10 days after hatching when the plants were at the three-leaf stage (V3). Five FAW larvae of the second to the third instar were deposited per plant for infestation. An artist’s paintbrush was used to transfer the larvae from the petri dish onto the flag leaf, the first fully formed leaf, for infestation. A second infestation was administered at the fourth vegetative growth stage (V4) using six third- to fourth-instar FAW larvae per plant. Eight days after the first infestation, a second infestation took place to ensure sustained pest pressure.

### 2.4. Data Collection

#### 2.4.1. FAW Larvae Survival and Developmental Stages

The numbers of surviving FAW larvae grown on the natural and artificial diets were recorded to determine the preferred diet. A FAW larva was considered to survive if it developed from hatching through all larval instars to successful pupation [21]. Data on FAW life cycle stages or metamorphosis, including the average duration, FAW morphological features and behaviour in terms of feeding and movement during each developmental stage, were documented. Average durations of each stage were obtained by observing seven independent egg batches of FAW at mean temperature and relative humidity conditions of 21 °C and 50% in the laboratory. For 15 selected FAW individuals within each egg batch, the average durations of each phase from the neonate to the adult moth stage were documented. Images of the salient life cycle stages were captured for comparison and reference under the test conditions.

#### 2.4.2. Reaction of Maize Genotypes to FAW

Maize genotypes were rated for FAW resistance. Resistance was assessed based on FAW damage scores obtained after the first and second infestations. The first scoring was conducted four days after the first infestation, and the following data were collected: fall armyworm leaf damage type and magnitude, number of leaves for each seedling plant and presence or absence of live FAW larvae and fresh frass on each plant. After the second infestation, FAW leaf-damage (FLD) rating was recorded at six-day intervals for four weeks. A 1 to 9 scale adapted from Davis et al. [39] for rating FAW damage was used, where a score of 1 represented a healthy plant with no damage symptoms and 9 represented a completely damaged plant with no possibility of recovery (Table 3). Continued leaf damage assessments that designated the FAW leaf damage as 1 to 5 (FLD1 to FLD5, corresponding to the level of FAW-inflicted leaf damage on the seedling plants that were obtained during the first to the fifth rating) were conducted by examining the damage on all the plants of each genotype and assigning an average score according to the rating scale up to the mid-whorl growth stage of the maize plants when foliar feeding was substantially reduced. Data on the type of damage were recorded considering the variations in FAW-inflicted damage on maize, ranging from no damage to damage restricted to the whorl, leaf or stalk and to a combination of whorl, leaf and stalk damage on the seedling plants.

### 2.5. Data Analysis

#### 2.5.1. Survival Rates of FAW Larvae under Two Contrasting Diets

Data on the numbers of surviving FAW larvae raised on the natural and artificial diets were converted to percentage survival rate. Comparisons between the mean numbers of surviving FAW larvae from the two diets were made by performing a paired-sample t-test in Genstat 18th edition [40].

#### 2.5.2. Developmental Stages of FAW under Laboratory Conditions

The average times taken, in days, to complete each FAW life cycle stage obtained through the monitoring of FAW individuals from seven egg batches (A to G) were subjected to analysis of variance (ANOVA) using Genstat 18th edition [40]. The mean durations for each life cycle stage across the independent egg batches and a mean duration for each stage were computed.

#### 2.5.3. Analysis of Variance on Leaf Damage Scores Mean Comparison Maize Genotypes

The Shapiro–Wilk test of normality was performed on the FAW-inflicted leaf damage data for the genotypes used in the study to determine the homogeneity of variances. Then, the leaf damage data were subjected to analysis of variance using GenStat [40] to discern the significant differences among genotypes for FAW-inflicted leaf damage.

#### 2.5.4. The Area under the Pest Progress Curve

The area under pest progress curve (AUPPC) was calculated based on FAW damage scores collected from the test genotypes during the early- to mid-whorl growth stages of the maize plants following Heinrichs and Miller [41] and Jeger and Viljanen-Rollinson [42]. AUPPC was computed as follows:

$$AUPPC=\overset{n=1}{\underset{i=1}{\sum }}\left[\left(\frac{FLD_{i}+FLD_{i+1}}{2}\right)\left(t_{i+1}-t_{i}\right)\right]$$

where

$\;FLD_{i\;}$ represents the mean of the ith fall armyworm leaf damage (FLD) across the three replications, beginning with FLD0 to FLD5
$\;Y_{i+1}$ represents the mean of the ith FLD plus 1
$t_{i\;}$ represents the ith time point at which leaf damage assessments were made, beginning with 14 days to 32 days after the first signs of FAW infestation
$\;t_{i+1}$ represents the ith time plus 1

Based on AUPPC, the genotypes were classified into top-, average- and bottom-performing groups. Low AUPPC is related to high genotype performance displaying low FAW damage scores.

## 3. Results

### 3.1. Survival Rates of FAW Larvae under Two Contrasting Diets

A paired-sample t-test analysis revealed that the survival rate of FAW larvae was significantly different (*p* < 0.001) between the two diets (Table 4). A higher mean larva survival rate of 80% was recorded for the natural rather than the artificial diet. Among the 8 sets, each initiated with 15 neonate FAW larvae grown on the natural diet, a mean of 12 larvae (80%) survived and developed from the larval instars and successfully pupated. For larvae raised on the artificial diet, a mean of six larvae (40%) survived and developed through the larval instars to pupation, suggesting that the FAW can quickly reproduce using a maize-based diet with no other special requirements.

### 3.2. Developmental Stages of FAW under Zambian Conditions

**Figure 1:**
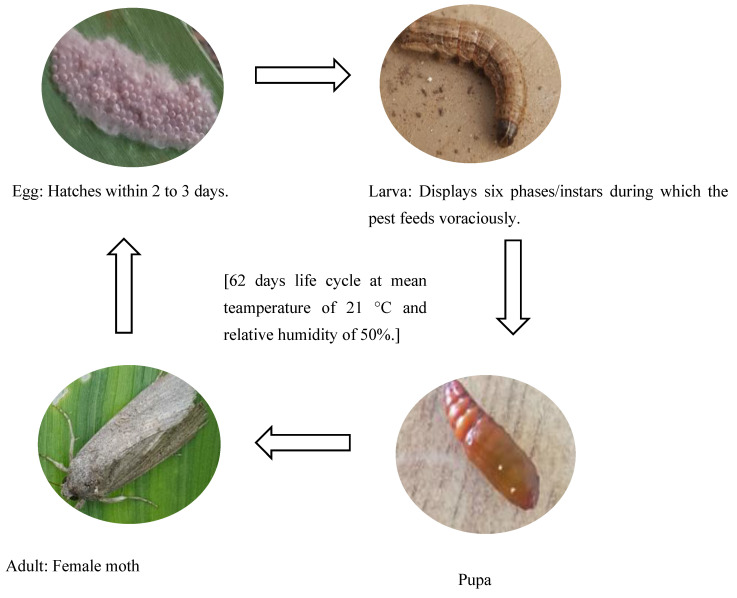
The life cycle of FAW with the egg, larva, pupa and adult stages.

The four FAW developmental stages (egg, larva, pupa and adult moth), the duration of each stage and their salient features are demarcated using the controlled reproduction of the pest in Zambia. This will guide the accurate identification of the pest and subsequent distinction from other related lepidopterans (Figure 1). Additionally, the sub-stages of the life cycle are recorded and summarized in Table 4.

The FAW’s egg, larval, pupa and moth stages had varying mean durations under the current test conditions. The larval stage, with six instars, was the longest and had a mean duration of 24 days. On average, the egg, pupa and moth stages lasted 2, 20 and 12 days, respectively (Table 5).

Within the larval stage, the third instar was the shortest, followed by the second and fourth instars, which had a similar duration. Important transitional phases related to the egg, larva and pupal stages were also identified (Table 6). The observed transitional phases included the progressive egg colour changes from the blackhead, ecdysis and prepupal phases, which depicted early and late transitions related to the egg, larval and pupal stages, respectively.

### 3.3. Selection of Maize Genotypes with FAW Resistance under Controlled Screening

#### 3.3.1. Analysis of Variance Based on Leaf Damage Scores

Analysis of variance revealed nonsignificant differences among the test genotypes for the first leaf damage score when infested with FAW larvae (Table 7). Differences among the genotypes were significant (*p* < 0.05) for the second leaf damage score and highly significant (*p* < 0.01) for the third, fourth and fifth leaf scoring.

#### 3.3.2. Mean Performance of Test Genotypes

Most test genotypes had FLD1 ratings below the score of 2. Only 6% of the genotypes had an FLD1 score of 3, while 30% had a score of 2 at FLD1. FAW damage scores for the genotypes were most variable at FLD3 followed by FLD4 (Table 8). The mean performance values for the all the genotypes in the study are recorded in Supplementary Table S2.

#### 3.3.3. Nature of FAW Damage and Reaction of Test Genotypes to Artificial FAW Infestation

The test genotypes showed damage characteristics in response to artificial infestation with FAW larvae. Damage characteristics ranged from 0 (no signs of FAW feeding) to 5 (visible leaf and whorl damage). The nature of the specific FAW-related damage observed in 15 selected genotypes is shown in Table 9. FAW-related damage profiles of all the 63 test genotypes are presented in Supplementary Table S3. Damage to the leaf only was the most common FAW-related symptom among the genotypes, while damage to the leaf and whorl was the next most common symptom of FAW damage. Stalk damage was the least common damage symptom observed among the genotypes.

#### 3.3.4. The Area under Pest Progress Curve (AUPPC)

**Figure 2:**
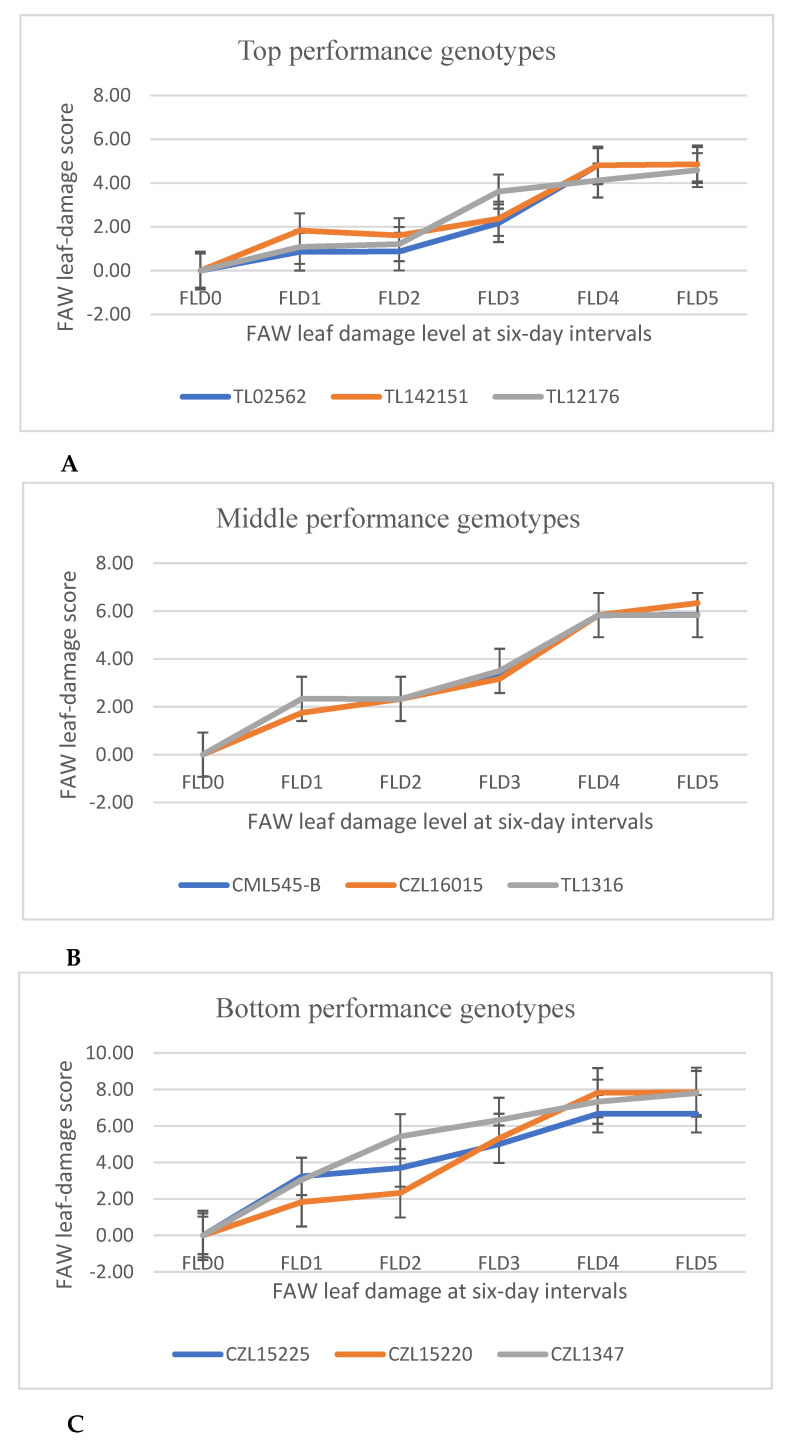
Pattern of damage progression for (**A**) top-performing, (**B**) middle-performing and (**C**) bottom-performing maize genotypes evaluated under artificial FAW infestation.

Table 10 and Figure 2a–c display the response patterns of the test genotypes to the FAW feeding damage. The FAW leaf damage ratings of the test genotypes gradually increased from 0.00 to 6.33 between FLD0 and FLD5 (Table 10). A relatively rapid increase in the FAW damage ratings was recorded between FLD0 and FLD1 and between FLD3 and FLD4.

The AUPPCs for the genotypes ranged from 61.63 to 137.94 (Supplementary Table S2), with TL02562 and CZL1347 having the lowest and highest values, respectively. The top-, middle- and bottom-performing genotypes showed clear damage progression trends, which increased from FLD0 to FLD4 and levelled off at FLD5 (Figure 2). The final leaf damage scores for the top-, middle- and bottom-performing genotypes were 4.86, 6.33 and 7.80, respectively.

## 4. Discussion

### 4.1. Rearing of FAW on the Natural vs. Artificial Diet

The present study successfully reared FAW in petri dishes using field-collected egg and larval stages and in oviposition cages using adult moths. Although the FAW clearly preferred the natural over the artificial diet, rearing on the artificial diet was still successful. This has implications for FAW rearing and genotype screening. This, in turn, enables the maintenance of constant FAW colonies for continued research. Considering the FAW is a polyphagous feeder, there are many artificial diets that can be used for rearing it under controlled conditions. Jin et al. [22] compared the performance of various diets for the rearing of FAW. The differences in performance of the diets suggest the possibility for optimization to suit particular laboratory conditions, which would increase the chances of FAW survival or success in rearing. Further studies are required in SSA to enable optimization of artificial diets for FAW rearing, infestation and development, and precision screening.

### 4.2. Developmental Stages of the FAW

The average durations of the FAW life cycle stages with the exception of the egg stage, differed from those of Montezano et al. [29], who reported means of 2.6, 13.73 and 9.24 days for the egg, larva and pupa stages, in that order. Deole and Paul [33] reported a life span of five to seven days for the FAW moth under field conditions, which was almost half the period observed under controlled conditions in this study. These differences were attributable to the laboratory conditions of temperature, relative humidity and photoperiod used in this study, which were different from those used in the other studies. In the current study, six larval instars were observed as documented in previous studies [29,33,43]. Although the laboratory conditions used in this study enabled the successful observation of all the life cycle stages, a few deviations were observed from what is currently known about FAW development in nature. For instance, oviposited eggs were mostly heaped rather than layered, as occurs naturally, and most of the cocoons appeared fragile compared with those collected from maize fields. The heaping of eggs in the laboratory may be attributed to the effect of the confinement of the female moths within the rearing cages. The fragile appearance could be associated with the absence of soil particles typically used by the insect for developing the pupal casing under field conditions [36].

Most freshly laid eggs collected from maize fields hatched within an average of two days, but a few egg batches did not hatch at all, probably due to unfavourable temperatures, parasitism or egg masses that trapped the neonates that failed to emerge successfully due to injury [35,36]. Egg masses gave rise to variable numbers of neonates that actively dispersed whenever the petri dish was opened by means of cobweb-like silk threads after a period of dormancy. In addition to dispersal, neonates and young larvae of the first and second instars are known to use the silk threads as a defence mechanism that helps them to drop rapidly to the ground whenever they are threatened [44].

The larval period, the longest of the life cycle stages (24 days in this study), is the feeding stage of the FAW, within which temperature and diet are very important factors for growth and development. López et al. [45] reported that FAW larvae required minimum temperatures ranging between 8 and 10 °C for survival in Mexico. In South Africa, the minimum temperature for FAW larval survival was reported to be 12 °C [35]. Santos et al. [20] observed rapid FAW larval growth and shorter instars under higher temperatures. In this study, the final instar took unusually long, and there is a possibility that a seventh instar may have occurred. He et al. [46] reported a FAW sixth instar length of 6.9 days and a seventh instar of 5.9 days, which together approximate the length of what was recorded as the maximum value for the final instar in this study. The possibility of a seventh instar having occurred is speculative and requires further investigation. Previous studies suggest the use of heat units or growth degree day units (GDDU) as a reliable means of determining the developmental rate of FAW [35,45].

The increased larval survival on the natural diet compared with the artificial diet corroborates the observation by Castro and Pitre [18], who recorded high larval mortality on soybean, one of the key ingredients of the artificial diet used in this study. Thus, a diet composed largely of maize extracts would be more suitable and affordable for raising the FAW in an insectarium in Zambia and similar agroecologies. Feeding during the larval stage progressively increases, with the first three instars observed to only skeletonize and punch small holes through maize leaves, while the fourth to sixth instars are responsible for more severe damage, including eating whole-leaf portions and destroying small plants. In FAW monitoring and surveillance, farmers should monitor their fields for skeletonized maize leaves, which would indicate the very first presence of FAW because very few lepidopteran species cause such damage [8,36]. The FAW larva is a gregarious feeder during its last instar stage, usually the sixth instar. Notably, a seventh instar sometimes occurs [47]. It is not known precisely what leads to the occurrence of a seventh instar, but the diet and environmental conditions may contribute. Instar stage changes can be determined by the careful monitoring of body colour, body length and head capsule width. The transition from one instar to the next is marked by ecdysis or moulting, a process through which the larva sheds its outer layer. Ecdysis would be a single, reliable means of identifying the change from one instar to the next except that the shedding of the outer skeleton is not easily seen in earlier instars because of their small size. In addition, ecdysis has implications for FAW control considering that moulted larvae are powerless and relatively easy to destroy mechanically or through the use of biocontrol agents.

Following the instar stage of rapacious feeding, the FAW larva moves into a slothful prepupal phase when it discontinues feeding [48]. In nature, the prepupal phase is not commonly observed because it usually occurs in the soil and lasts only for a maximum of three days under warm conditions [33]. The laboratory rearing of FAW offers the opportunity to view the prepupal phase and to observe its features before the actual pupation occurs. Substantial numbers of FAW in the prepupal stage were lost during their transition to pupa and during pupal growth, resulting in a marked reduction in the numbers of FAW moths. Although the duration of pupation observed in this study was 20 days, previous studies have reported a maximum pupation period of 45 days [36]. Under field conditions, pupation mostly occurs underground, when prepupal larvae burrow in the soil to a depth of 25 to 75 mm. In this study, pupation experienced the highest mortality, with the pupal casing of dead pupae assuming a dark brown to black appearance such as that observed immediately before the emergence of the FAW moth.

The genders of adult FAW moths were easily distinguishable by their colour patterns, with female moths being dull coloured compared with the male moths [33,34,36]. Being nocturnal, the FAW were dormant during the day and only moved when persistently agitated. They were observed to feed and lay eggs late at night. This is in agreement with reports by Luginbill [36], who found that laboratory-reared FAW moths laid eggs after midnight. Both male and female moths were observed to lose vitality progressively. Successful mating and oviposition were observed in the rearing cages, but counts of egg batches were not conducted because of the limited number of surviving moths following high levels of mortality at pupation.

### 4.3. Artificial Infestation of Maize Genotypes

The nonsignificant differences exhibited by the test genotypes for the first FAW leaf damage assessment (FLD) could be attributed to the uniform nature of feeding damage caused by first to third instar larvae that were inoculated on the maize plants. Leaf-damage assessments during the FLD3 to FLD5 revealed significant differences among the test genotypes. This may be because the assessments were conducted at a time when the inoculated larvae had developed into differentially advanced instars in response to the differences among the host maize genotypes. In addition, later assessments were conducted when the host–pest interaction was sufficiently established to trigger the inherent plant-defence mechanisms whose intensity would depend largely on the genetic background of the host plant [49]. The most variable leaf-damage ratings showed up at FLD3, suggesting that the best time to detect differences in FAW leaf damage among the test genotypes under the given experimental conditions was at three weeks after the first infestation. However, to capture differences more accurately, repeated assessments during vegetative growth are recommended. The progression of FAW damage substantially reduced between FLD4 and FLD5 to an almost constant horizontal trend (Table 10). This trend may be attributed to the FAW’s known behaviour of abandoning its feeding on the leaves at later whorl stages to strategically position itself in the position of the emerging ear shoots and tassels. Emerging ear shoots and tassels become visible at this stage of the plant’s growth (V6) when magnification is used [8,49,50]. A more detailed investigation of the profiled genotypes and similar germplasm is required to enhance our understanding of maize responses to FAW feeding.

The defined set of damage characteristics in response to FAW feeding has useful implications for pest management. Farmers are encouraged to closely monitor their fields for pest damage symptoms, which constitute many of the symptoms identified in this study. The high frequency of leaf and whorl damage symptoms observed in this study agrees with the findings of Abrahams et al. [1], who reported that foliar damage is the most typical FAW-related damage symptom in maize.

The damage profiles of the test genotypes enabled the identification of promising genotypes in terms of damage severity for further breeding. Tropical maize inbred lines, including TL13159, TL02562, TL142151, VL050120 and CML548-B, exhibited resistance reactions, whereas CML545-B, CZL1310c, CZL16095, EBL169550 and open-pollinated varieties ZM4236 and Pool 16 displayed moderate resistance to the FAW and were all selected for advanced evaluation (Table 11). FAW preferences for these genotypes were low (Pool 16), moderate (TL13159, TL142151, CML548-B, CML545-B, CZL1310c, CZL16095, ZM4236) and high (EBL169550) when previously screened under natural FAW infestation by Kasoma et al. [32]. Therefore, the selected maize genotypes are recommended as sources of FAW resistance and should be evaluated under multiple and representative growing environments for breeding or large-scale production. The baseline information presented in this paper will allow for reliable FAW infestation, genotype screening and the integration of candidate FAW resistance genes into market-preferred maize lines in Zambia and related agroecologies.

In conclusion, this study determined the salient features of FAW growth and development under local controlled conditions. This will assist plant breeders in undertaking controlled host–plant resistance screening and enhance breeding efforts. The study also identified candidate maize genotypes for the validation of FAW resistance and other farmer-preferred traits under field conditions of FAW infestation. Subsequently, these genotypes can be used to develop suitable germplasm to be incorporated in the development of a coherent IPM program for FAW management in Zambia and similar agroecologies.

## Figures and Tables

**Table 1:** The head capsule widths of FAW larval instars observed under controlled laboratory conditions of 25° C and 70% relative humidity (Montezano et al. 2019 [29]).

Instar	Mean Head Capsule Width (mm)
FIRST	0.35 ± 0.02
Second	0.56 ± 0.03
Third	0.87 ± 0.04
Fourth	1.27 ± 0.06
Fifth	1.85 ± 0.12
Sixth	2.72 ± 0.20

**Table 2:** Descriptions of maize genotypes selected for the study.

Genotype	Breeding History	Source	Presumed FAW Resistance *	Genotype	Breeding History	Source	Presumed FAW Resistance *	Genotypes	Breeding History	Source	Presumed FAW Resistance *
CKDHL0323	Inbred line	CIMMYT	MR	CZL15225	Inbred line	CIMMYT	MR	Teost	OPV	NPGRC	-
CML441-B	Inbred line	CIMMYT	MR	CZL15123	Inbred line	CIMMYT	MR	TL101711	Inbred line	CIMMYT	-
CML488	Inbred line	CIMMYT	MR	CZL15231	Inbred line	CIMMYT	S	TL102562	Inbred line	CIMMYT	-
CML491	Inbred line	CIMMYT	S	CZL15234	Inbred line	CIMMYT	MR	TL116163	Inbred line	CIMMYT	MR
CML538	Inbred line	CIMMYT	MR	CZL16015	Inbred line	CIMMYT	S	TL118367	Inbred line	CIMMYT	-
CML539	Inbred line	CIMMYT	S	CZL16016	Inbred line	CIMMYT	MR	TL12176	Inbred line	CIMMYT	MR
CML545-B	Inbred line	CIMMYT	MR	CZL16080	Inbred line	CIMMYT	S	TL13159	Inbred line	CIMMYT	MR
CML546-B	Inbred line	CIMMYT	S	CZL16084	Inbred line	CIMMYT	MR	TL1316	Inbred line	CIMMYT	MR
CML547-B	Inbred line	CIMMYT	S	CZL16091	Inbred line	CIMMYT	MR	TL139113	Inbred line	CIMMYT	-
CML548-B	Inbred line	CIMMYT	MR	CZL16093	Inbred line	CIMMYT	S	TL139180	Inbred line	CIMMYT	MR
CML572	Inbred line	CIMMYT	MR	CZL16095	Inbred line	CIMMYT	MR	TL142017	Inbred line	CIMMYT	R
CZL03011	Inbred line	CIMMYT	S	CZL16098	Inbred line	CIMMYT	MR	TL142139	Inbred line	CIMMYT	MR
CZL052	Inbred line	CIMMYT	MR	CZL16137	Inbred line	CIMMYT	S	TL142151	Inbred line	CIMMYT	MR
CZL1310c	Inbred line	CIMMYT	MR	CZL16141	Inbred line	CIMMYT	MR	TL14266	Inbred line	CIMMYT	-
CZL1347	Inbred line	CIMMYT	S	EBL1611480	Inbred line	CIMMYT	-	TL145748	Inbred line	CIMMYT	-
CZL1369	Inbred line	CIMMYT	MR	EBL169550	Inbred line	CIMMYT	R	TL1512847	Inbred line	CIMMYT	S
CZL1466	Inbred line	CIMMYT	MR	EBL173782	Inbred line	CIMMYT	-	TL1512845	Inbred line	CIMMYT	MR
CZL15033	Inbred line	CIMMYT	MR	EBL1738809	Inbred line	CIMMYT	-	TL173	Inbred line	CIMMYT	MR
CZL15142	Inbred line	CIMMYT	S	MM501	Hybrid	ZAMSEED	S	VL05120	Inbred line	CIMMYT	-
CZL15209	Inbred line	CIMMYT	MR	MM502	Hybrid	ZAMSEED	MR	ZM4236	OPV	NPGRC	MR
CZL15220	Inbred line	CIMMYT	MR	Pool 16	OPV	ZAMSEED	S	ZM7114	OPV	NPGRC	MR

CIMMYT = International Maize and Wheat Improvement Center; OPV = Open Pollinated Variety; ZAMSEED = Zambia Seed Company Limited; NPGRC = National Plant Genetic Resources Center; - = not available ; R = resistant; MR = moderately resistant; S = susceptible; * FAW reaction based on field evaluation of genotypes by Kasoma et al. (2020) [31].

**Table 3:** Rating scale used to score maize genotypes artificially infested with FAW larvae in Zambia (Adapted from Davis, Ng & Williams, 1992 [39]).

Symptom Description	Score
No visible damage	1
2–4 windowpane-damaged portions	2
2–4 windowpane-damaged portions and 2–4 pin/shot holes	3
5–10 windowpane-damaged portions and shot holes	4
10–15 windowpane-damaged portions and shreds only	5
10–15 windowpane-damaged portions shot holes and shreds	6
10–15 windowpane-damaged portions, shot holes, shreds and traces of whorl damage	7
≥15 windowpane-damaged portions, shot holes, shreds and moderately damaged whorl	8
≥15 windowpane-damaged portions, shot holes, shreds and completely damaged whorl	9

**Table 4:** Rate of FAW larval survival (% proportion) and significant tests under natural and artificial diets.

Set	Diet	
	Natural	Artificial
1	11 (73.3%)	4 (26.7%)
2	13 (86.7%)	8 (53.3%)
3	9 (60.0%)	7 (46.7%)
4	15 (100.0%)	5 (33.3%)
5	12 (80.0%)	6 (40.0%
6	13 (86.7%)	6 (40.0%)
7	11 (73.3%)	10 (66.67%)
8	12 (80.00%)	2 (13.3%)
t-statistics, (df = 7)	5.15	
Significance level	0.000662	
Standard deviation (SD)	1.77	2.45
Mean	12 (80.00%)	6 (40.00%)
Minimum	9	2
Maximum	15	10

Note: A natural diet contains fresh maize leaves and stalks, while an artificial diet consists of processed flour from soybean, wheat germ and other vital ingredients. Individual larvae in each of the eight sets were place singly in separate petri dishes.

**Table 5:** Observed durations of FAW life cycle stages under laboratory conditions.

Egg Batch ID	Life Cycle Stages										Number of FAW for Observation	
	Egg	L1	L2	L3	L4	L5	L6	Pre-Pupa	Pupa	Adult	Initial Number at Neonate Stage	Number at Pupal Stage
A	2.0	2.0	3.6	3.6	1.4	2.3	12.2	4.7	17.7	7.0	15.0	5.0
B	3.0	3.0	2.9	3.2	2.7	2.7	13.4	3.6	19.7	23.0	15.0	8.0
C	-	2.8	2.7	2.7	2.7	3.4	11.3	4.3	19.8	5.0	15.0	13.0
D	2.3	-	2.9	2.9	2.9	2.2	9.8	3.0	22.0	7.0	15.0	13.0
E	2.2	2.8	2.8	2.8	2.8	-	10.8	2.8	25.6	2.0	15.0	15.0
F	2.0	2.9	2.6	3.0	2.7	2.6	11.0	3.4	18.2	17.3	15.0	9.0
G	2.7	2.0	2.0	3.0	3.4	2.6	5.4	2.8	15.0	20.3	15.0	4.0
Mean	2.4	2.6	2.8	3.0	2.7	2.6	10.6	3.5	19.7	11.7	15.0	9.6
Standard deviation	0.9	1.0	1.0	1.1	1.0	1.0	2.8	1.3	4.3	21.6	5.3	5.0
Minimum	2.0	2.0	2.0	2.7	1.4	2.2	5.4	2.8	15.0	2.0	15.0	4.0
Maximum	3.0	3.0	3.6	3.6	3.4	3.4	13.4	4.7	25.6	23.0	15.0	15.0
F-statistic	130.85 ***	1.42 ^ns^	13.63 ***	4.09 **	17.23 ***	2.04 ^ns^	3.20 **	2.21 *	2.63 *	-	-	-
SE	0.13	0.45	0.39	0.40	0.40	0.79	3.21	1.42	5.76	-	-	-
CV (%)	5.6	15.9	13.8	13.9	15.1	29.7	30.0	40	31.5	-	-	-

L1–L6 = first to the sixth larval instars of FAW; ns = not significant; *, **, *** = significant at *p* < 0 05, *p* < 0.01 and *p* < 0.001 respectively; SE = standard error; CV = coefficient of variation. - = data not available for the duration of the moth stage because of the high mortality of FAW individuals at this stage.

**Table 6:** Description of the developmental stages of FAW on maize leaf under controlled laboratory conditions in Zambia.

Stage	Features	Sub-Stage	Descriptions	Duration (Days)	Appearance
Egg	With three sub-stages distinguishable by colour changes from green to cream white to black.	I	Eggs are covered by scales from the female moth. They appear green to grey for 12 h and begin to darken.	<1	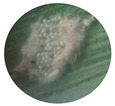
		II	Cream-white to pink, transitioning into brown.	1	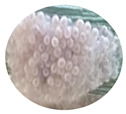
		III	Dark egg mass approaching hatching. Egg mass appears grey to black before hatching.	1	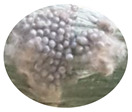
Egg–larva	Immobile, circular, and shiny black protrusions in fur-like mass	Eclosion	Black shiny heads are visible as they emerge from the eggshells.	<1	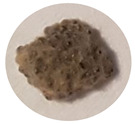
Larva	Feeding stage: with six substages distinguishable by body colour changes and feeding patterns	Blackhead	Newly hatched larvae on a tender maize stalk remain dormant for about 5 to 6 h.	<1	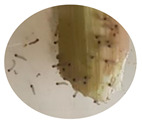
		Instar I	First instar larvae on a maize leaf. Small bodies and shiny blackheads.	2–3	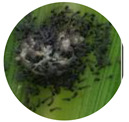
		Instar II	Second instar larva on a maize leaf with a cream to pale white body and blackhead.	2–4	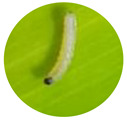
		Instar III	Third instar larva: light brown, begins to turn green after feeding on leaves.	3–4	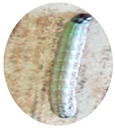
		Instar IV	Fourth instar larva: dark green or dark brown body.	1.5–3	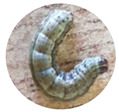
		Instar V	Fifth instar larva: well defined brown body with an inverted Y marking on the head.	2–3	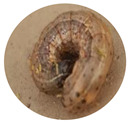
		Instar VI	Sixth instar larva surrounded by frass. Grey or dark brown body with fully defined segments and head markings.	5–13	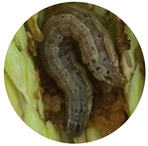
	Progressive shedding of the exoskeleton with a pale colour prior to the darkening of the body	Ecdysis/moulting	Larva sheds its outer cuticular skeleton between instars, leaving a colourless patch in the neck area. Ecdysis lasts approximately 12 to 28 h.	>1	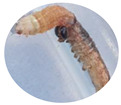
	Dark skeleton	Ecdysis/moulting	The skeleton appears black.	>1	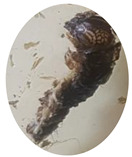
Larva–Pupa	Inactive and compacted body	Pre-pupa	It stops feeding, and the body becomes short, preparing for pupation. Body segments with defined ridges and markings.	3–5	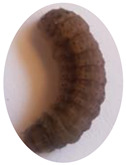
Pupa	Stiff pupal casing with localized circular movements in the head area of the insect	Consists of the early, mid, and late pupal stages	Forms an oval-shaped cocoon using leaf particles. The cocoon gradually changes from green to pink to orange-brown.	15–26	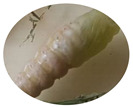
Adult	Dull-coloured moth	No distinguishable sub-stages	Female is comparatively plain in appearance with no prominent marks on the wings.	12	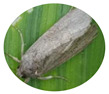
			Male moth has conspicuous markings on wings.		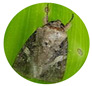

**Table 7:** Analysis of variance for FAW leaf damage among 63 genotypes evaluated under artificial FAW infestation in the screenhouse.

Source	df	FLD1	FLD2	FLD3	FLD4	FLD5
Replications	2	0.2979	1.604	5.748	8.8314	5.0628
Genotypes	62	0.9604	6.334 *	3.398 **	1.505 **	1.3976 **
Residual	100	0.8583	4.39	2.191	0.9676	0.8671

DF = Degrees of freedom; *, ** = significant at *p* < 0.05 and *p* < 0.01 respectively; FLD1 refers to the first FAW leaf damage rate recorded 4 days after the first infestation; FLD2, FLD3, FLD4 and FLD5 refer to the second, third, fourth and fifth FAW leaf-damage rates recorded at six-day intervals after the second infestation.

**Table 8:** Mean performance of the top, middle and bottom five maize genotypes based on lowa damage scores.

Genotype	FAW Leaf-Damage Rates							
	FLD0	FLD1	FLD2	FLD3	FLD4	FLD5	Mean FLD	AUPPC
	Top five performing genotypes							
TL02562	0.00	0.86	0.87	2.17	4.81	4.86	2.26	61.63
TL142151	0.00	1.83	1.61	2.37	4.81	4.86	2.58	67.29
TL12176	0.00	1.08	1.21	3.61	4.12	4.59	2.44	67.39
TL13159	0.00	2.33	1.25	1.83	5.83	5.83	2.85	70.98
Teost	0.00	1.33	1.75	2.00	5.50	5.67	2.71	72.50
	Middle five performing genotypes							
EBL169550	0.00	1.50	1.50	3.17	6.17	6.83	3.20	85.52
Pool 16	0.00	1.33	2.09	4.29	4.60	6.82	3.19	86.33
CML545-B	0.00	2.33	2.33	3.32	5.82	5.85	3.28	86.39
CZL16015	0.00	1.75	2.33	3.17	5.83	6.33	3.24	87.00
TL1316	0.00	2.33	2.33	3.50	5.83	5.83	3.30	87.48
	Bottom five performing genotypes							
CML547-B	0.00	2.91	2.84	4.00	7.00	7.33	4.01	105.04
CZL16141	0.00	1.92	1.72	5.78	6.76	6.82	3.83	106.04
CZL15225	0.00	3.24	3.70	5.00	6.67	6.67	4.21	112.20
CZL15220	0.00	1.83	2.33	5.32	7.82	7.85	4.19	116.39
CZL1347	0.00	3.05	5.43	6.33	7.33	7.80	4.99	137.94
	Statistics							
Grand mean	0.00	1.85	2.05	3.26	6.05	6.33	3.35	87.33
CV (%)	-	49.60	92.60	45.60	16.30	14.80	-	-
LSD (0.05)	-	1.50	3.40	2.40	1.60	1.51	-	-
SED	-	0.93	2.10	1.48	0.98	0.93	-	-

FLD0 = stage prior to FLD1 with no visible FAW damage symptoms; FLD1 refers to the first FAW leaf damage rate recorded at 4 days after the first infestation; FLD2, FLD3, FLD4, FLD5 refer to the first, second, third, fourth and fifth FAW leaf-damage rates recorded at six-day intervals after the second infestation; AUPPC = area under pest progress curve; CV = coefficient of variation; LSD = least significant difference; SE = standard error of the mean difference; - = not applicable.

**Table 9:** FAW damage type and magnitude assessed from 15 representative maize genotypes evaluated under artificial FAW infestation.

Damage Type							
Genotype	None	Whorl Only	Leaf Only	Stalk	Leaf/Whorl and Fresh Frass	Leaf and Whorl	Number of Plants at the Final Assessment
CML545-B	0	0	5	0	0	1	6
CZL1466	0	2	0	0	1	1	4
CML491	0	0	3	0	0	0	3
CZL0310c	0	0	2	0	0	3	5
CML539	0	0	5	1	0	0	6
CZL15142	0	0	0	0	0	2	2
VL050120	0	0	1	0	0	0	1
CZL16095	0	0	1	0	0	1	2
ZM4236	0	0	0	0	0	3	3
EBL1611480	0	1	4	0	0	0	5
EBL169550	0	0	0	0	1	5	6
EBL173782	1	0	1	0	1	0	3
MM501	0	0	3	0	1	1	5
Pool 16	1	0	1	0	0	1	3
TL142151	0	0	1	0	0	0	1
Total number of plants showing the damage *	3	7	101	1	14	80	206

Note: The numbers 0–5 in the columns represent specific damage scores for the genotypes. The “number of plants at the final assessment” in the eighth column refers to the number of surviving plants at FLD5, the last damage rating performed on the plants. * refers to the total across all 63 genotypes, and each genotype was represented by 6 plants across 3 replications (2 plants per replication).

**Table 10:** FAW damage ratings based on AUPPCs for all maize genotypes from FLD1 to FLD5.

FAW Assessment Level	FAW Leaf Damage Score
FLD1	1.87
FLD2	2.26
FLD3	3.24
FLD4	6.03
FLD5	6.28

**Table 11:** Mean damage scores and FAW reactions for promising genotypes identified in the study.

Genotype	Mean FAW Damage Score	FAW Reaction
TL13159	3.41	Resistance
TL02562	2.71	Resistance
VL050120	3.49	Resistance
CML548-B	3.13	Resistance
CML545-B	3.92	Moderate resistance
CZL1310c	3.93	Moderate resistance
CZL16095	3.97	Moderate resistance
EBL169550	3.83	Moderate resistance
ZM4236	4.18	Moderate resistance
Pool 16	3.82	Moderate resistance

## Data Availability

All data are provided in the manuscript.

## Supplementary Materials

The following supporting information can be downloaded at: https://www.mdpi.com/article/10.3390/insects13111020/s1: Table S1: Artificial diet used for laboratory rearing of FAW. Table S2: Mean performance and AUPPCs of 63 tropical maize genotypes when evaluated under artificial FAW infestation. Table S3: Nature and magnitude of FAW damage revealed by 63 tropical maize genotypes evaluated under artificial FAW infestation. Supplementary Figure S1: Diets used for rearing FAW on petri dishes. S1A- Natural diet of maize leaves and stalks. S1B- Artificial diet containing wheat, soy and other ingredients. Supplementary Figure S2: Rearing cage for adult FAW moths.
