# Supplementary material for: Fall Armyworm Infestation and Development: Screening Tropical Maize Genotypes for Resistance in Zambia
Source: Insects. 2022 Nov 4;13(11):1020. doi: 10.3390/insects13111020 (PMC9694902; doi:10.3390/insects13111020)
Supplement: Supplementary file 1 [file insects-13-01020-s001.zip › Figures S1 and S2.pdf]

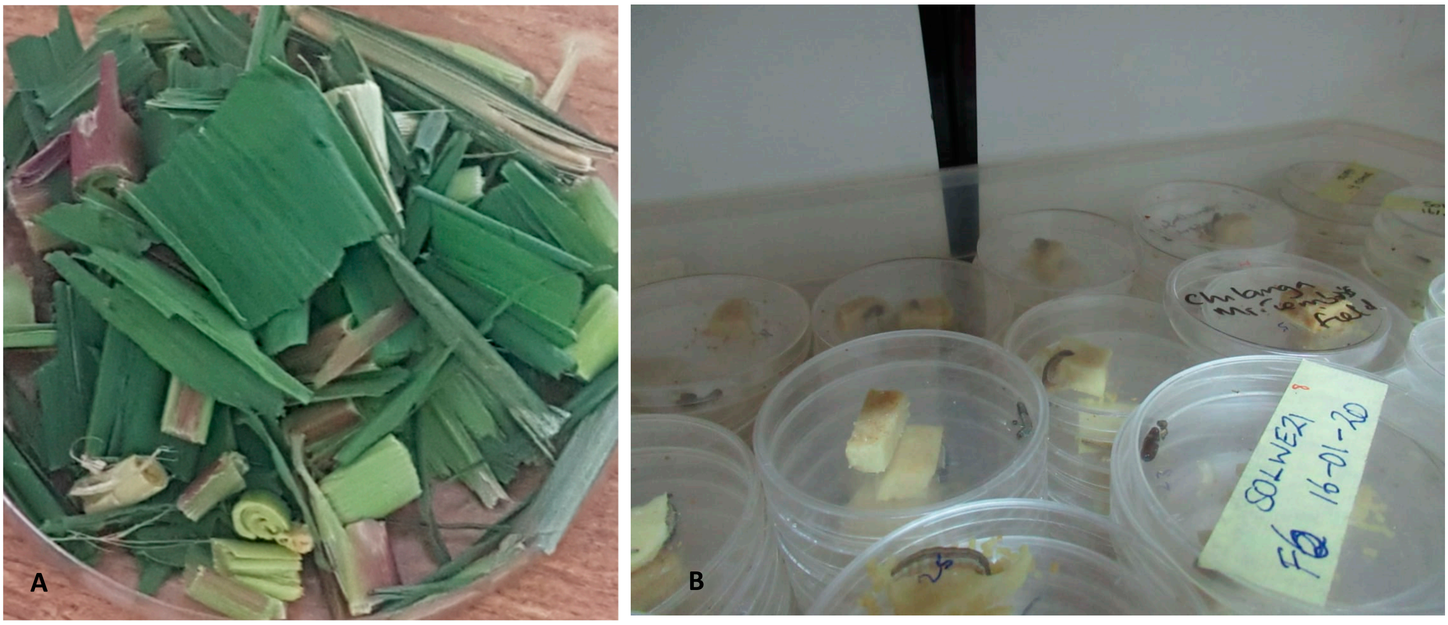

Figure S1: Diets used for rearing of FAW on Petri dishes. A- Natural diet of maize leaves and stalks. B- Artificial diet containing wheat, soy and other ingredients

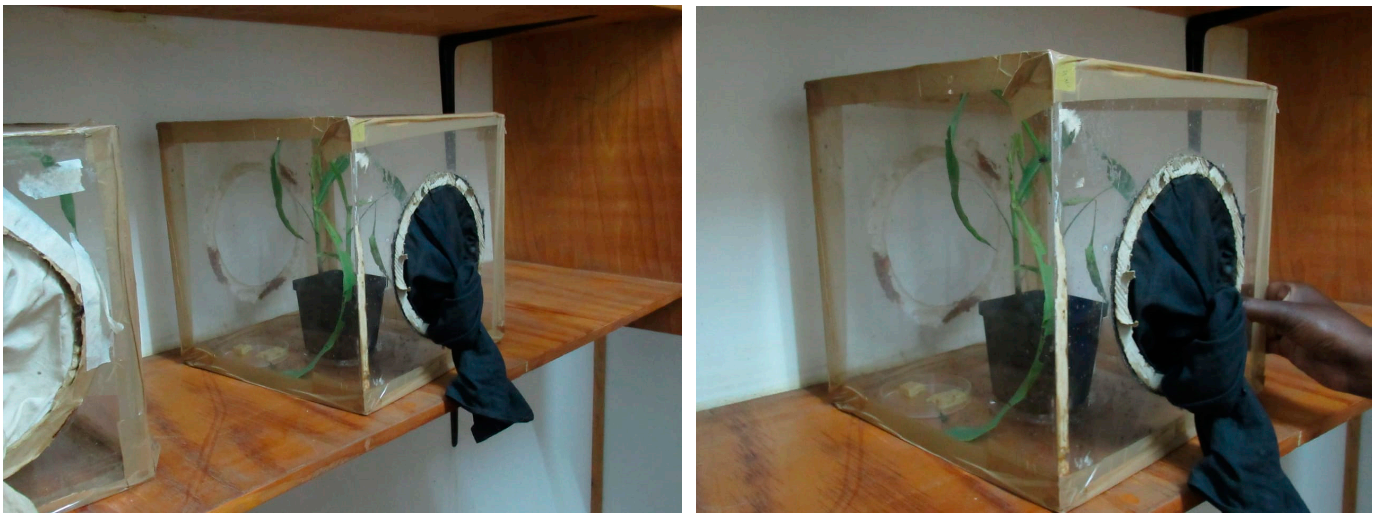

Figure S2: Rearing cage for adult FAW moths
