# Supplementary material for: Fall Armyworm Infestation and Development: Screening Tropical Maize Genotypes for Resistance in Zambia
Source: Insects. 2022 Nov 4;13(11):1020. doi: 10.3390/insects13111020 (PMC9694902; doi:10.3390/insects13111020)
Supplement: Supplementary file 1 [file insects-13-01020-s001.zip › Tables S1, S2 S3.pdf]

Table S1: Artificial diet used for laboratory rearing of FAW

Ingredient	Quantity (gram)	Apparatus	Ingredients of the dry mix
Agar	28.50	Microwave	Soybean flour
Dry mix	216.00	Blender	Sucrose (50%)
Antibiotic	1.50	Spatula	Wheat germ
Procedure			Salt mix
1: Add agar to 875ml of water			USDA† vitamin pre mix
2: Bring to boil for 1 minute			Fibre
3: Transfer agar solution to blender containing the dry mix			Methyl parabene, Sorbic acid
4: Blend for 30 seconds			Ascorbic acid
5: Dispense immediately			
6: Store diet in the refrigerator			

† USDA = United States Department of Agriculture

Table S2: Mean performance and AUPPC values of 63 tropical maize genotypes when evaluated under artificial FAW infestation

Name	FLD0†	FLD1‡	FLD2§	FLD3	FLD4	FLD5	AUPPC
TL02562	0.00	0.86	0.87	2.17	4.81	4.86	61.63
TL142151	0.00	1.83	1.61	2.37	4.81	4.86	67.29
TL12176	0.00	1.08	1.21	3.61	4.12	4.59	67.39
TL13159	0.00	2.33	1.25	1.83	5.83	5.83	70.98
Teost	0.00	1.33	1.75	2.00	5.50	5.67	72.50
CML548-B	0.00	1.25	1.25	2.00	5.67	6.67	73.50
TL14266	0.00	2.08	1.83	2.00	5.67	5.67	73.98
CZL15231	0.00	1.83	2.33	3.17	4.33	5.17	74.50
CZL052	0.00	1.33	1.33	2.50	5.83	5.83	75.48
CZL1369	0.00	1.10	1.22	2.83	5.78	5.84	76.52
CZL15234	0.00	1.83	2.00	2.33	5.67	5.67	76.98
CML539	0.00	2.17	2.50	2.83	5.00	5.17	77.48
CZL16137	0.00	1.58	1.92	2.00	6.00	6.00	77.52
VL050120	0.00	1.33	1.33	3.17	5.33	6.33	77.99
TL151845	0.00	1.33	1.67	1.67	6.33	6.67	78.04
TL139113	0.00	1.67	2.33	2.33	5.67	5.67	78.96
MM502	0.00	1.97	1.58	2.86	5.83	5.83	79.14
ZM7114	0.00	1.87	2.42	2.58	5.33	5.83	79.50
CZL16080	0.00	1.50	1.50	2.00	6.50	6.50	79.50
EBL1611480	0.00	1.42	1.42	2.83	6.00	6.00	79.50
TL142017	0.00	1.83	1.83	2.67	6.00	6.00	81.00
TL139180	0.00	1.83	2.17	2.67	5.67	6.00	81.04
CZL16084	0.00	1.75	2.33	2.36	5.88	6.37	82.54
CZL15209	0.00	2.25	2.67	2.67	5.67	5.67	83.04
CZL16093	0.00	1.58	1.83	2.83	6.17	6.17	83.46

CML491	0.00	3.02	2.87	3.11	5.31	5.36	83.78
TL118367	0.00	1.29	1.50	3.00	6.50	6.50	85.50
TL101711	0.00	0.78	2.17	2.83	6.17	6.17	85.50
EBL169550	0.00	1.50	1.50	3.17	6.17	6.83	85.52
Pool 16	0.00	1.33	2.09	4.29	4.60	6.82	86.33
CML545-B	0.00	2.33	2.33	3.32	5.82	5.85	86.39
CZL16015	0.00	1.75	2.33	3.17	5.83	6.33	87.00
TL1316	0.00	2.33	2.33	3.50	5.83	5.83	87.48
TL173	0.00	1.25	1.33	3.50	6.50	6.50	87.48
CML488	0.00	2.16	2.59	3.23	5.84	5.87	87.56
CZL16095	0.00	1.50	2.00	2.33	6.67	7.33	87.98
CZL16098	0.00	0.83	0.83	3.50	6.67	7.33	87.98
CZL16091	0.00	1.54	1.46	2.61	7.09	7.12	88.31
CML546-B	0.00	2.02	1.86	3.37	5.80	7.85	89.75
EBL173783	0.00	1.50	1.75	3.50	6.50	6.50	90.00
TL116163	0.00	1.50	2.67	3.33	6.00	6.00	90.00
CML572	0.00	2.27	2.37	2.61	6.62	6.85	90.14
CZL1310c	0.00	0.99	0.95	3.57	7.07	7.10	90.86
CKDHL0323	0.00	2.67	2.67	4.00	5.67	5.88	91.67
CZL1466	0.00	1.67	1.67	2.67	7.33	7.33	92.04
CML538	0.00	0.99	0.95	4.17	6.83	6.83	92.22
EBL1738809	0.00	2.33	2.83	2.83	6.50	6.50	92.46
CZL1523	0.00	2.04	1.96	3.61	6.59	6.62	92.81
ZM4236	0.00	2.08	2.83	3.83	5.83	6.33	93.95
CZL16016	0.00	2.25	2.75	3.58	6.17	6.33	93.98
TL145748	0.00	2.33	2.08	3.50	6.83	6.83	94.98
CZL15142	0.00	2.67	2.75	4.17	5.83	6.17	95.02
CZL15033	0.00	3.05	2.81	3.84	6.33	6.43	97.15
TL1412139	0.00	1.53	2.58	4.58	5.83	6.50	97.46
TL1512847	0.00	1.33	2.75	4.50	5.83	6.50	98.00
MM501	0.00	1.92	1.58	4.17	7.17	7.17	99.00
CZL03011	0.00	2.50	3.00	4.33	6.33	6.67	101.98
CML441-B	0.00	2.50	3.42	4.67	5.83	7.00	104.54
CML547-B	0.00	2.91	2.84	4.00	7.00	7.33	105.04
CZL16141	0.00	1.92	1.72	5.78	6.76	6.82	106.04
CZL15225	0.00	3.24	3.70	5.00	6.67	6.67	112.20
CZL15220	0.00	1.83	2.33	5.32	7.82	7.85	116.39
CZL1347	0.00	3.05	5.43	6.33	7.33	7.80	137.94
GM	0.00	1.85	2.09	3.26	6.05	6.33	87.33
CV (%)	-	49.60	92.60	45.60	16.30	14.80	-
LSD (0.05)	-	1.50	3.40	2.40	1.60	1.51	-
SE	-	0.93	2.10	1.48	0.98	0.93	-

† FLD0 = stage prior to FLD1 with no visible FAW damage symptoms; ‡ FLD1 = refers to the first FAW leaf damage rate recorded 4 days after the first infestation; § FLD2, FLD3, FLD4, FLD5 = refer to the first, second, third, fourth and fifth FAW

leaf-damage rates recorded at six-day intervals after the second infestation; ¶ AUPPC = Area Under Pest Progress Curve; †† GM = grand mean; ‡‡ CV = coefficient of variation; §§ LSD = least significant difference; ¶¶ SE = standard error

Table S3: Nature and magnitude of FAW damage revealed by 63 tropical maize genotypes evaluated under artificial FAW infestation

	Damage characteristic						
Genotype	None	Whorl only	Leaf only	Stalk	Leaf/whorl and fresh frass	Leaf and whorl	Number of plants at final assessment
CKDHL032	0	0	1	0	0	5	6
CML441-B	0	2	0	0	0	2	4
CML488	0	0	3	0	0	0	3
CML491	0	0	1	0	0	1	2
CML538	0	0	0	0	0	2	2
CML539	0	0	5	1	0	0	6
CML545-B	0	0	5	0	0	1	6
CML546-B	0	0	1	0	0	1	2
CML547-B	0	0	1	0	1	0	2
CML547B	0	0	2	0	0	0	2
CML548-B	0	0	0	0	0	2	2
CML572	0	0	1	0	0	0	1
CZL03011	0	0	4	0	0	1	5
CZL052	0	0	4	0	0	0	4
CZL1310c	0	0	2	0	0	3	5
CZL1347	0	0	1	0	0	2	3
CZL1466	0	2	0	0	1	1	4
CZL15033	0	0	1	0	2	0	3
CZL15142	0	0	0	0	0	2	2
CZL15209	0	0	3	0	0	2	5
CZL15220	0	0	1	0	0	2	3
CZL15225	0	0	3	0	1	1	5
CZL1523	0	0	0	0	1	1	2
CZL15231	0	1	0	0	0	2	3
CZL15234	0	0	5	0	0	1	6
CZL16015	0	0	2	0	1	1	4
CZL16016	0	0	2	0	1	2	5
CZL16080	0	0	2	0	1	0	3
CZL16084	0	0	0	0	1	1	2
CZL16091	0	0	3	0	0	0	3
CZL16093	0	0	3	0	0	0	3
CZL16095	0	0	1	0	0	1	2
CZL16098	0	0	1	0	0	2	3
CZL16137	0	0	5	0	0	0	5
CZL16141	0	0	0	0	0	2	2
EBL1611480	0	1	4	0	0	0	5

EBL169550	0	0	0	0	1	5	6
EBL173782	1	0	1	0	1	0	3
EBL1738809	0	0	2	0	0	2	4
MM501	0	0	3	0	1	1	5
MM502	0	0	0	0	0	1	1
Pool16	1	0	1	0	0	1	3
Teost	0	0	1	0	1	1	3
TL101711	0	0	1	0	0	0	1
TL102562	0	0	1	0	0	0	1
TL116163	0	1	2	0	0	0	3
TL118367	0	0	1	0	0	1	2
TL13159	0	0	0	0	0	2	2
TL1316	0	0	3	0	0	2	5
TL139113	0	0	1	0	0	1	2
TL139180	0	0	1	0	0	3	4
TL142017	0	0	1	0	0	2	3
TL142139	0	0	2	0	0	4	6
TL142151	0	0	1	0	0	0	1
TL14266	0	0	3	0	0	2	5
TL145748	0	0	4	0	0	2	6
TL151284	0	0	0	0	0	1	1
TL151845	1	0	1	0	0	1	3
TL173	0	0	2	0	0	4	6
VL05120	0	0	1	0	0	0	1
ZM4236	0	0	0	0	0	3	3
ZM7114	0	0	1	0	0	0	1
Total number of plants showing the damage	3	7	101	1	14	80	206
